# A Comprehensive Review on the Enhancement Mechanism of Fatigue Performance in Titanium Alloys via Laser Shock Peening

**DOI:** 10.3390/nano16050321

**Published:** 2026-03-03

**Authors:** Qun Zu, Jiong Yang, Jiarui Li, Xinxin Qi, Xiao Yang

**Affiliations:** 1 School of Mechanical Engineering, Hebei University of Technology, Tianjin 300401, China; 2 Key Laboratory of Hebei Province on Scale-Span Intelligent Equipment Technology, Tianjin 300401, China

**Keywords:** laser shock peening, nanostructure behavior, titanium alloy, fatigue property

## Abstract

This paper reviews the enhancement mechanisms of fatigue performance in titanium alloys processed by laser shock peening (LSP). Because of the redistribution of residual stress and micro-crack and pore behavior, micro–nanostructure evolution and surface roughness effect are systematically discussed. LSP induces beneficial compressive residual stresses at the surface, effectively suppressing crack initiation and propagation. Notably, the nanostructures induced by this process—including nanotwins, dislocations, stacking faults, and nanocrystals—collectively enhance the material’s mechanical hardness, strength, and fatigue resistance. Furthermore, LSP reduces porosity, alters pore morphology and alters crack initiation sites, thereby increasing the crack propagation threshold. However, the influence of LSP on material toughness remains a subject of debate. The insights provided herein offer valuable theoretical guidance for the development of high-performance titanium alloys and further optimization of LSP technology.

## 1. Introduction

Titanium and its alloys have been widely utilized as structural materials in aerospace, marine, automotive, and biomedical applications owing to their excellent strength-to-weight ratio, corrosion resistance, and thermal stability [1,2,3]. However, the severe service environments involving sustained cyclic loading under elevated temperatures and pressures necessitate enhancing the fatigue resistance of materials to ensure structural integrity and operational safety [4,5,6,7]. Statistical analysis indicates that fatigue-induced failures constituted ~60% of component failures in aircraft structures [8] and frequently result in catastrophic consequences and substantial economic losses.

Surface strengthening technologies are the most effective approach for enhancing fatigue resistance, with established techniques including shot peening, surface rolling and low-plasticity burnishing. However, these methods have obvious limitations and are inadequate for meeting the increasingly stringent performance and precision requirements of materials. For example, SP induces detrimental surface roughness and demonstrates inadequate geometric adaptability to complex components [9,10]. The surface rolling process faces the challenge of achieving optimal surface integrity of materials [11], while low-plasticity burnishing risks surface embrittlement [12].

**Figure 1:**
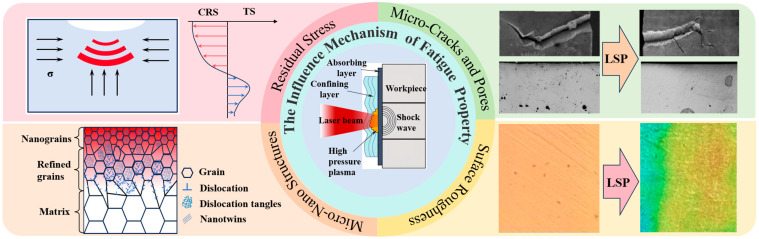
Schematic diagram of the principle of laser shock peening and the mechanism of fatigue performance enhancement in titanium alloys.

Recently, the laser shock peening (LSP) has evolved into an advanced surface modification technique. Distinct from the conventional approaches, LSP can contribute to the deeper compressive residual stress layers, better process controllability and more stable strengthening effects [13], thereby enhancing the fatigue resistance. The underlying principle, as illustrated in Figure 1, involves a high-power-density, short-pulse laser passing through a transparent confining medium to irradiate a target surface coated with an energy-absorbing layer [14]. This interaction generates confined plasma with extreme temperatures and pressures. The resulting shock wave propagates into the material once the transient pressure exceeds the dynamic yield strength, inducing gradient plastic deformation and consequent redistribution of residual stresses along the depth direction [15].

To enable efficient implementation and optimization of laser shock peening, this review systematically elucidates the mechanisms by which LSP enhances the fatigue performance of titanium alloys. To ensure comprehensiveness and objectivity, a systematic literature search was conducted in English-language databases, including Web of Science and Scopus, for relevant studies published since 2000. The search employed Boolean operators to combine keywords such as (“laser shock peening” OR “laser shock processing”) AND (“titanium alloy”) AND (“fatigue” OR “crack growth” OR “nanoscale structure” OR other related terms), focusing on studies investigating the mechanisms underlying fatigue property improvement. Following a systematic screening process, the eligible literature was thoroughly summarized, leading to the identification of four main factors governing the influence of LSP on the fatigue performance of titanium alloys: (i) residual stress redistribution; (ii) micro-crack and pore evolution; (iii) micro–nanostructure evolution; and (iv) surface morphology effect.

## 2. Residual Stress

Residual tensile stresses are commonly generated near the surface of titanium alloys during both subtractive and additive manufacturing (AM) processes, resulting from thermal gradients, mechanical forces, etc. [16,17]. Under cyclic loading conditions, these tensile residual stresses can act as driving forces for cracks, thereby accelerating fatigue crack propagation rates [18]. LSP utilizes high-energy-laser-induced shock waves to induce surface compression and plastic deformation, which produces a residual compressive stress field along the shock wave propagation direction [19]. The presence of compressive residual stress field effectively suppresses fatigue crack initiation, retards crack propagation, mitigates stress concentrations, and consequently enhances the overall fatigue performance of titanium alloys [20]. The influence of residual stress $\sigma^{r}$ on fatigue limit can be described by the Goodman relationship [21,22], as expressed in Equation (1):

(1) $$\Delta \sigma^{r}=-m\sigma^{r}$$

where $\Delta \sigma^{r}$ represents the variation in the fatigue limit caused by residual stress (All strength-related quantities in the equations that follow are expressed in MPa), $m=\sigma_{a0}/\sigma_{tf}$ is the mean stress sensitivity coefficient, $\sigma_{a0}$ is the fatigue strength at zero static stress, and $\sigma_{tf}$ is the true fracture strength. According to the relationship, the residual tensile stress ($\sigma^{r}$ > 0) diminishes the fatigue limit, whereas the residual compressive stress ($\sigma^{r}$ < 0) elevates it. Typically, a higher magnitude and greater uniformity of compressive residual stress, combined with an increased depth of the affected layer, lead to significantly enhanced fatigue performance in materials [23,24].

**Figure 2:**
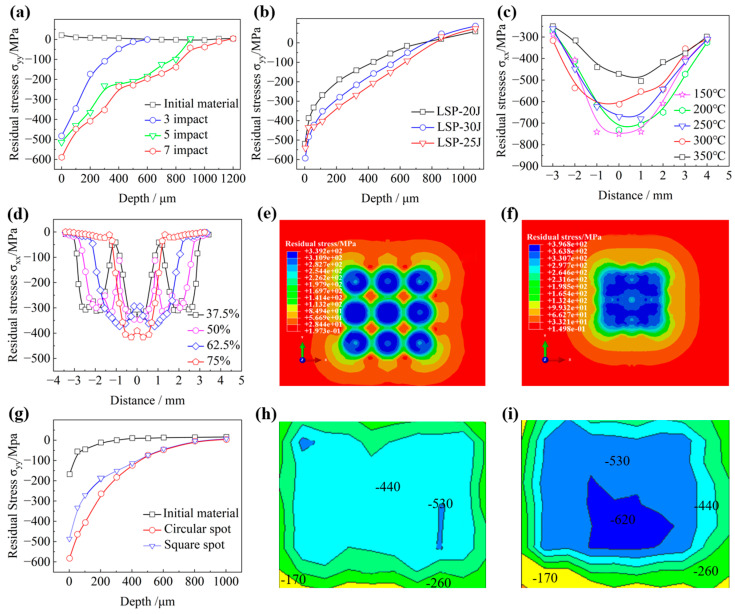
LSP parameter effect on the induced compressive residual stress in titanium alloys: (**a**) laser energy; (**b**) impact times; (**c**) temperature; (**d**–**f**) overlap ratio; and (**g**–**i**) laser spot shape. Reprinted with permission from [27,28,29,30,31] (Note: The residual stresses presented were characterized by X-ray diffraction (XRD), except for those in (**d**–**f**), which were obtained from numerical simulations).

The residual compressive stress layer generated by LSP can exceed 1 mm, approximately 2~5 times greater than those achievable via conventional shot peening (SP) [25]. This enhancement effect is governed by multiple laser parameters, including impact count, pulse energy, spot overlap ratio, spot geometry, etc., as systematically illustrated in Figure 2. Generally, both residual stress magnitude and penetration depth increased with higher pulse energy [26] and increased impact counts [27]; while, some experimental observations confirmed that supra-optimal energy levels could reduce the residual compressive stress (Figure 2b) [28]. Furthermore, the thermal stability study on TC4 titanium alloy by Pan et al. [29] revealed that the peak compressive stress exhibited a monotonic decrease as the temperature increased, undergoing accelerated degradation above 300 °C (Figure 2c). The elevated overlap ratios promoted the enhanced stress-field uniformity and greater depth efficiency, as shown in Figure 2d–f [30]. By contrast, the circular spots generated higher peak stresses, whereas square configurations achieved superior stress homogeneity (Figure 2g–i) [31].

**Figure 3:**
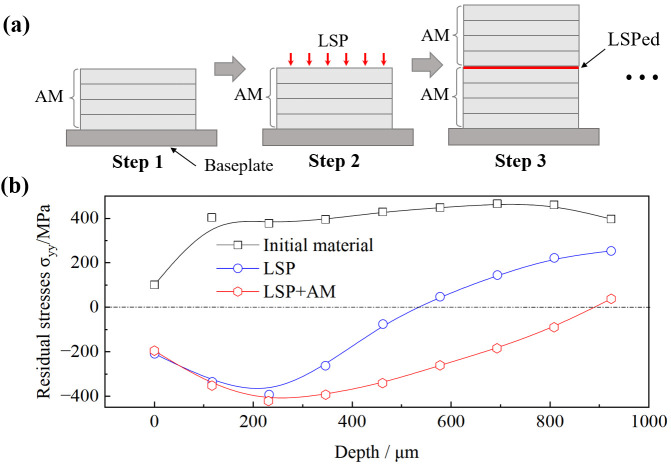
(**a**) Hybrid processing schematic of LSP and AM, (**b**) residual stress distribution in TC4 samples under different processing conditions. Reprinted with permission from [33].

Nevertheless, the capability of introducing compressive residual stresses through LSP alone is fundamentally limited. To overcome this constraint, “LSP+” hybrid processing strategies have been developed to induce the residual stresses with broader spatial coverage, enhanced uniformity and superior controllability. Representative implementations include “LSP + SP” [32], “LSP + AM” [33], “LSP + dynamic aging” [34], etc. Luo et al. [32] reported that the components with substantial surface compressive stresses and significantly deepened stress-affected zones were obtained through implementing of “LSP + SP”. Kalentics et al. [33] implemented a layer-by-layer strategy wherein LSP was applied at predetermined layer intervals during AM (Figure 3a). Comparative analysis demonstrated this synergistic approach delivered notably enhanced peak compressive stresses and greater penetration depths relative to conventional LSP processing (Figure 3b).

## 3. Micro-Cracks and Pores

During the manufacturing, forming and service stages of titanium alloys, defects such as cracks and pores inevitably occur [16,17]. Under cyclic loading, these defects act as initiation sites for fatigue cracks, thereby compromising structural integrity and reliability [35]. The existing research has revealed that LSP significantly enhances fatigue performance by modifying crack and pore behavior through four primary mechanisms of reducing porosity, altering the pore morphology, relocating the crack nucleation site and decreasing the crack propagation rate.

### 3.1. Reducing Porosity

**Figure 4:**
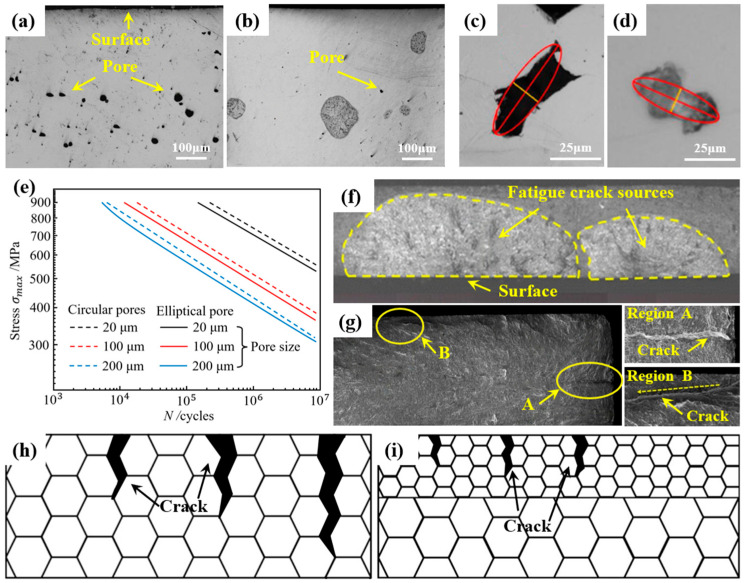
Micro-crack/pore evolution and fatigue life of titanium alloys in as-built and LSP-processed samples: (**a**,**b**) cross-sectional pore porosity [36]; (**c**,**d**) surface pore morphology [39]; (**e**) S-N fatigue life curves [40]; (**f**,**g**) fatigue fracture surface morphology [42]; (**h**,**i**) schematic illustration of crack propagation [37]. All the figures were reprinted with permission.

Tong et al. [27] observed a significant reduction in the number of micro-cracks on the corrosion layer surface of TC11 titanium alloy following LSP treatment. Similarly, Chen et al. [36] investigated cross-sectional pores in titanium nanocomposites subjected to varying impact cycles (Figure 4a,b). The results showed that LSP could effectively reduce the porosity adjacent to the LSP surface, decreasing the pore volume fraction from 1.71% to 0.12% with a reduction of nearly 93%. The effectiveness of LSP in improving porosity is highly dependent on the initial size, shape, depth, and surface connectivity of the pores. The enhanced fatigue performance is attributed principally to two mechanisms based on the porosity: (i) Compressive residual stresses induced by LSP compress and close micro-cracks in the vicinity of the laser shock-affected zone, thereby delaying crack initiation [37,38]; (ii) Reduced pore volume fraction increases the effective load-bearing area and enhances mechanical strength.

### 3.2. Altering Pore Morphology

Comparative analysis by Kalentics et al. [39] revealed a 68% reduction in average pore size within the LSP-affected zone relative to untreated specimens, accompanied by a transition toward more circular pore geometries (Figure 4c,d). This morphological modification mitigated stress concentration, resulting in a more than 15-fold increase in flexural fatigue life and a 44% improvement in fatigue limit compared to untreated samples. Furthermore, Bergant et al. [40] used a short-crack propagation model to predict S-N curves for pores of various geometries and dimensions (Figure 4e). It was observed that components with circular pores exhibited extended fatigue life, whereas larger or elongated pores degraded the fatigue resistance.

### 3.3. Relocating Crack Initiation Location

Wang et al. [41] investigated the mechanisms by which LSP influenced the fatigue behavior of TC4 titanium alloy. It was found that fatigue cracks in LSP-treated specimens preferentially initiated at subsurface locations, unlike surface sites observed in untreated material. As shown in Figure 4f,g, the untreated samples exhibited numerous surface-initiated fatigue cracks with pronounced striations and secondary cracking, featuring a high local stress concentration. In contrast, LSP samples displayed fewer initiation sites which were distributed in subsurface regions, contributing to the improved fatigue performance [42].

### 3.4. Decreasing Crack Propagation Rates

Typically, the crack initiation occurs at grain boundary triple junctions, followed by propagation along grain boundaries until arrest at the next junction. Further crack advancement requires additional energy consumption to overcome this barrier [37]. As illustrated in Figure 4h,i, LSP induces grain refinement, which increases the density of triple junctions. The microstructural modification necessitates higher energy dissipation during crack propagation, thereby effectively suppressing crack initiation. Furthermore, the refined grains within the LSP-affected zone exhibit larger misorientation angles [43]. This enhanced misorientation impedes the dislocation movement and improves the dislocation pile-up at grain boundaries. These combined effects increase the number of cycles required for crack nucleation in the LSP-affected zone, effectively raising the crack growth threshold and reducing the crack propagation rate [44].

## 4. Micro–Nano Structures

**Figure 5:**
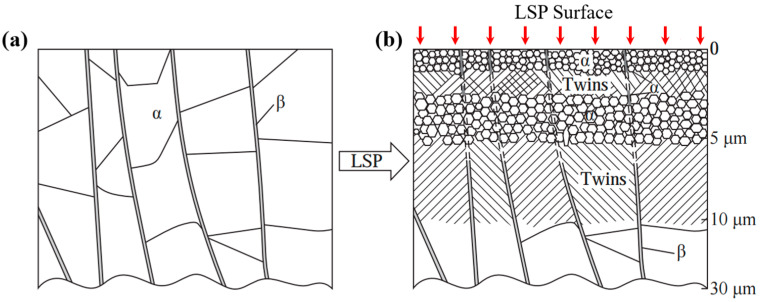
Schematic diagram of the microstructural evolution from (**a**) as-prepared EBM sample to (**b**) LSP-treated EBM sample. Reprinted with permission from [45].

**Figure 6:**
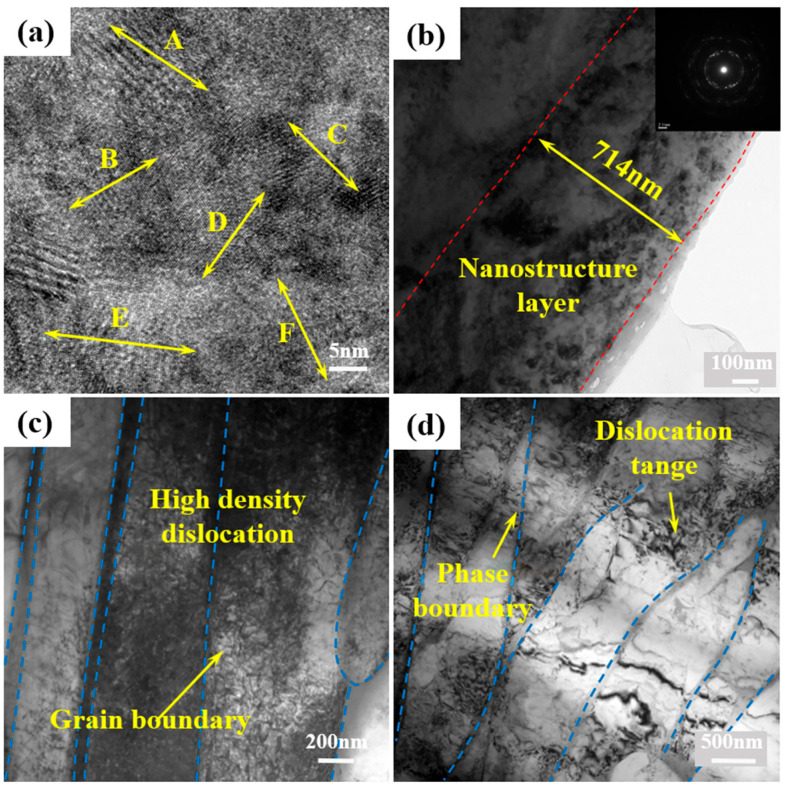
TEM morphology of the microstructure at different depths after LSP: (**a**) surface nanocrystals; (**b**) nanocrystal layer; (**c**) high-density dislocations; (**d**) low-density dislocations. Reprinted with permission from [46].

The micro–nanostructure evolution induced by LSP processing in titanium alloys plays a crucial role in enhancing fatigue performance. A high strain rate and high-energy loading typically induce grain refinement from the microscale to the nanoscale, accompanied by the formation of high-density dislocations and nanoscale twins, as well as pronounced dislocation tangling and interactions with interfaces. Jin et al. [45] conducted comparative microstructural analysis of electron-beam-melted (EBM) TC4 titanium alloy in both as-fabricated and LSP-treated conditions. The significant microstructural transformation is explained in Figure 5. The EBM-formed alloy exhibited a characteristic equilibrium α + β dual-phase structure. Following LSP treatment, this layered structure evolved into a gradient microstructure comprising equiaxed nanocrystals, deformation twins, and submicron α-phase grains. Consequently, the fatigue limit increased from 600 MPa to 700 MPa, representing a ~16.7% enhancement. Figure 6 presents the transmission electron microscopy (TEM) morphology of TC4 titanium alloy along the impact direction after laser shock processing at 3.6 J, as reported by Wang et al. [46]. Figure 6a shows the nanocrystalline structure at the shocked surface, where regions marked A–F correspond to nanograins with different crystallographic orientations and an average grain size of 10 nm. Figure 6b displays the nanocrystalline region within approximately 1 μm from the shocked surface, with a thickness of about 710.4 nm. Figure 6c and Figure 6d illustrate the areas at depths of 50 μm and 100 μm from the surface, exhibiting high-density and low-density dislocations, respectively, indicating a gradual decrease in dislocation density with increasing depth. Along with these graded microstructural changes, the high-cycle fatigue strength of the sample increased from 210 MPa to 255 MPa, representing an enhancement of approximately 21.43% compared to the unshocked specimen.

Qu et al. [47] investigated the fatigue enhancement mechanism of LSP-treated Ti17 alloys, attributing the improvement primarily to gradient structures characterized by high dislocation densities, deformation twins, and stacking faults. These structural features contributed to a remarkable 197% increase in fatigue life compared to untreated specimens. Similarly, Wu et al. [48] demonstrated that high dislocation density suppressed the motion of mobile dislocations toward the free surface, delayed crack initiation, and increased the energy required for micro-crack propagation, thereby reducing crack growth rate and extending fatigue life.

Nanostructural parameters, such as dislocation density, grain size, etc., can directly affect the mechanical properties of titanium alloys, particularly including hardness, strength and toughness [49,50]. The quantitative correlation between the microstructure and mechanical properties remains a central research focus in materials science.

### 4.1. Relationship of Fatigue Strength, Micro-Hardness and Nanostructure

The empirical relationship between the fatigue strength and micro-hardness of metallic materials has been expressed by Equation (2) [49,51]:

(2) $$\sigma_{w}=1.6HV\pm 0.1HV(HV\leq 400)$$

where $\sigma_{w}$ is the fatigue strength and HV is the hardness (All hardness values involved in the subsequent equations refer to Vickers hardness and are expressed in ${\mathrm{kgf}/\mathrm{mm}}^{2}$). The hardness of conventional titanium alloys typically ranges from 250 HV to 350 HV, while LSP-treated surfaces rarely exceed 400 HV [52,53]. Equation (2) indicates that fatigue strength increases approximately proportionally with micro-hardness. The hardened surface improves wear resistance, mitigates foreign object damage and suppresses surface crack initiation, collectively contributing to enhanced fatigue performance [29,54].

**Figure 7:**
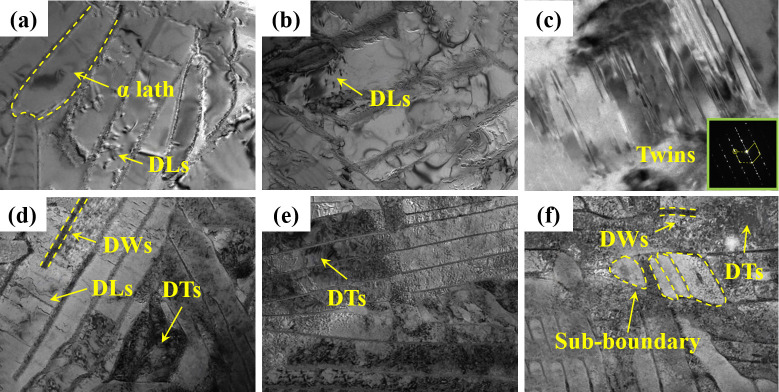
TEM microstructural morphology of TC4 titanium alloy: (**a**,**b**) as-built sample; (**c**–**f**) LSP-treated sample. Reprinted with permission from [56].

Chen et al. [55] reported that micro-hardness variation along the treated surface directly corresponded to microstructural gradients. As the laser pulse propagated and attenuated within the substrate, the extent of grain refinement progressively diminished, leading to a corresponding reduction in micro-hardness. Chi et al. [56] analyzed the LSP-affected layer via TEM, elucidating the mechanism of laser-induced nanograins. In untreated TC4 alloys, only sparse dislocation lines (DLs) were observed (Figure 7a,b). After LSP treatment, numerous nanoscale twins appeared in the α phase due to the limited slip systems of the hexagonal close-packed (HCP) lattice (Figure 7c). Subsequently, twin boundaries facilitated the formation of stacking faults, which generated high-density DLs (Figure 7d). Progressive dislocation accumulation led to the formation of dislocation tangles (DTs) and dislocation walls (DWs) (Figure 7e). Ultimately, the continued dislocation rearrangement during laser shocking induced subgrain boundary formation (Figure 7f), resulting in pronounced grain refinement.

**Figure 8:**
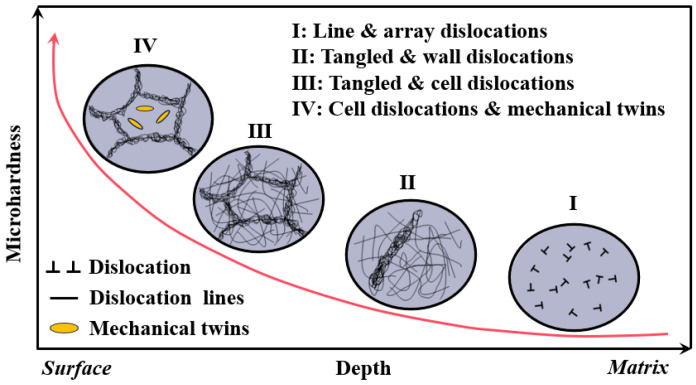
Schematic diagram of the relationship between micro-hardness and microstructure. Reprinted with permission from [57].

Zhang et al. [57] categorized the micro-hardness evolution after LSP into four stages (Figure 8). Stages I-II corresponded to dislocation-mediated strengthening, where LSP-induced plastic deformation generated and accumulated the dislocations. Stage III represents grain refinement strengthening, characterized by subgrain boundary formation that progressively refined grains and impeded dislocation slip. Stage IV involved twinning-dominated strengthening, where numerous nanoscale twins near the surface were generated, further increasing the hardness. Collectively, these stages demonstrate that the enhancement of micro-hardness is governed by dislocation-mediated strengthening, grain refinement, and nanotwin strengthening [58].

According to the Taylor strengthening model, the relationship between micro-hardness and dislocation density *ρ* is given by Equation (3) [59]:

(3) $$HV=HV_{0}+3\alpha M^{T}Gb\rho^{1/2}$$

where $HV_{0}$ accounts for the hardness contributions coming from other sources than dislocation-dislocation interaction, *α* is a parameter describing the strength of the interaction between dislocations, $M^{T}$ is the Taylor factor, *G* is the shear modulus, *b* is the Burgers vector and *ρ* is the dislocation density. Equation (3) indicates that the material hardness scales proportionally with the square root of dislocation density.

According to the Hall–Petch relationship, the inverse proportionality between material micro-hardness and grain diameter is as follows [60]:

(4) $$HV=HV_{1}+kd^{-1/2}$$

where $HV_{1}$ captures hardness except grain refinement, *k* is the Hall–Petch coefficient and *d* is the average grain diameter. Through molecular dynamics simulations, Niu et al. determined that the critical grain size for polycrystalline titanium is 19.96 nm. When the average grain size exceeds the critical value, the model follows the Hall–Petch relationship [61].

LSP promotes the formation of numerous nanotwins near the subsurface layer of titanium alloys. Their strengthening effect can be described as [56]

(5) $$HV=HV_{2}+fk_{y}t^{-1/2}$$

where $HV_{2}$ represents hardness in addition to nanotwin interactions, *f* is the nanotwin volume fraction, *t* is the average twin thickness, and $k_{y}$ is the Hall–Petch coefficient. As Equation (5) shows, HV increases proportionally with the ratio $t^{-1/2}$, thus laser shock-induced nanotwin significantly enhances the micro-hardness of titanium alloys.

### 4.2. Relationship of Fatigue Strength, Tensile Strength, and Nanostructure

Based on the work of Wöhler et al. [62], Pang et al. [49] proposed a correlation between the fatigue strength $\sigma_{w}$ and tensile strength $\sigma_{b}$ of metallic materials:

(6) $$\sigma_{w}=(C-P\sigma_{b})\sigma_{b}$$

where *C* and *P* are material constants. For titanium alloys, C=0.62 and $P=1.09\times 10^{-4}\;{\mathrm{MPa}}^{-1}$. Calculations show that when $\sigma_{b}<2844\;\mathrm{MPa}$, the fatigue performance of titanium alloys increases with tensile strength. Generally, the tensile strength of LSP-treated titanium alloys typically does not exceed 2844 MPa. Therefore, higher strength in titanium alloys typically translates to greater load-bearing capacity and superior fatigue performance.

**Figure 9:**
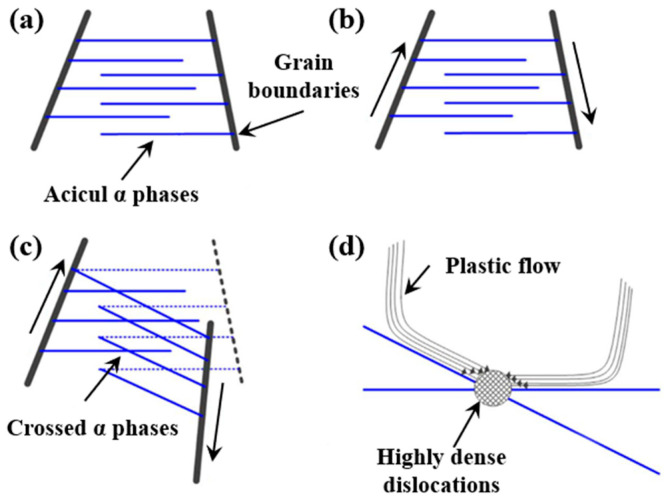
Schematic illustration of LSP-induced formation of high-density dislocations: (**a**) as-built sample with acicul α phases; (**b**) intense relative movement of grain boundaries under LSP; (**c**) numerous α phases intersection; (**d**) dense dislocations formation at the intersections of α phases. Reprinted with permission from [65].

Lan et al. [63] reported a 3.9% increase in the yield strength of TC4 alloy following LSP treatment. This improvement was attributed to *α*-phase refinement caused by laser-induced shock waves, while the increased number of grain boundaries effectively impeded dislocation motion and hindered dislocation transmission across grains. Guo et al. [64] found that high-density dislocations and multi-directional nanoscale twins generated by LSP were the dominant factors contributing to enhanced yield and ultimate tensile strength in TC4 titanium alloys. Meng et al. [65] demonstrated that high-energy laser shocks promoted intense grain boundary activity, causing rotation and intersection of needle-like *α* phases. This process induces localized plastic strain accumulation and the formation of high dislocation densities (Figure 9), which restrict further plastic deformation and increase yield strength.

According to the Hall–Petch strengthening model, parallel nanotwins shorten dislocation free paths and introduce additional interfaces that impede dislocation movement, thus enhancing overall strength [66]. Similarly, Taylor hardening theory suggests that high dislocation densities contribute to strength improvement [67]. Therefore, the increased strength observed after LSP originates from synergistic contributions of micro–nanostructure evolution, predominantly including grain refinement, deformation twinning and dislocation accumulation.

Grain refinement increases the density of grain boundaries, which obstructs dislocation motion. The corresponding Hall–Petch relationship is given by [68]

(7) $$\sigma_{NG}=k_{y}d^{-1/2}$$

where $\sigma_{NG}$ is the grain refinement-induced strength. As Equation (7) indicates, $\sigma_{NG}$ increases as *d* decreases.

Morever, LSP generates high-density dislocation networks within the subsurface region. The Taylor hardening model expresses the contribution of dislocation structures as [66]

(8) $$\sigma_{D}=M^{T}\alpha Gb\rho^{1/2}$$

where $\sigma_{D}$ is the dislocation-induced strength, and Equation (8) shows that $\sigma_{D}$ is proportional to $\rho^{1/2}$, confirming that a higher dislocation density leads to greater material strength.

Although the definitive theoretical relationship between twin density and tensile strength remains ambiguous, the experimental results have demonstrated that the mechanical twins generally elevate material strength [69]. Yokoyama et al. [70] proposed that deformation twinning and grain boundary plasticity were strongly coupled during deformation, dynamically reconfiguring hierarchical twin boundary and grain boundary networks, and the dense networks of deformation twins endowed metals with unprecedented mechanical properties.

### 4.3. Relationship Between Fatigue Strength, Toughness, and Nanostructure

A definitive quantitative correlation between fatigue strength and toughness in titanium alloys remains unestablished. According to the study by Ye [71], the fracture toughness characterized a material’s resistance to deformation and crack propagation under impact loading, thereby alleviating stress concentration effects during fatigue cycling. Empirically, materials with superior fracture toughness typically exhibit enhanced fatigue resistance.

**Figure 10:**
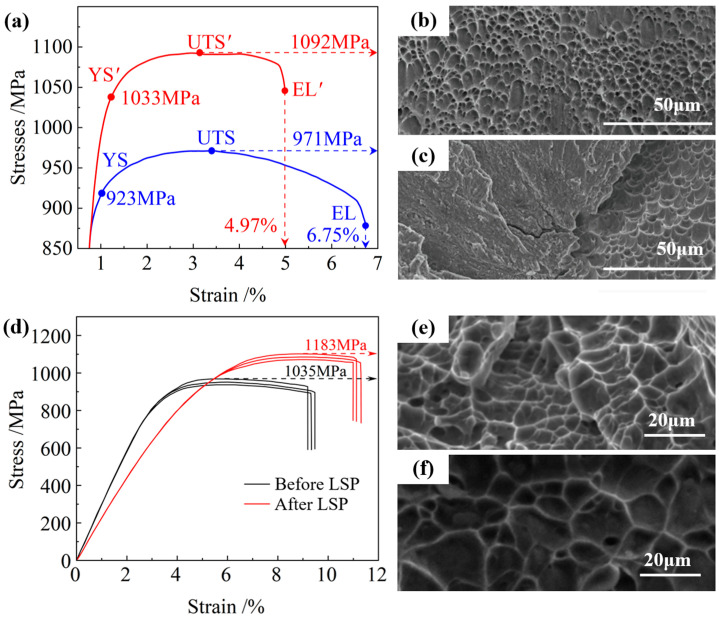
Tensile stress–strain curves in (**a**) TA15 [56] and (**d**) TC4 [75]. Typical SEM fracture morphologies of TC4 titanium alloy in (**b**,**e**) as-built sample, (**c**,**f**) LSP sample [64,73]. All the figures were reprinted with permission.

Typically, the strength enhancement accompanies with compromised fracture toughness for most metallic systems [72]. Consequently, while LSP increases strength, it may reduce toughness. Chi et al. [56] investigated the stress–strain behavior of TA15 titanium alloy before and after LSP treatment, as depicted in Figure 10a. Although LSP increased the yield strength, it concurrently reduced the elongation by approximately 26%. Yang et al. [73] analyzed the fracture morphology of TC4 titanium alloy, observing deep equiaxed dimples and micro-voids in the untreated sample (Figure 10b). In contrast, LSP-treated samples predominantly exhibited flatter cleavage facets, indicating reduced toughness (Figure 10c).

Zhang et al. [74] suggested that under the ultrahigh-strain-rate impact of LSP, excessively dense dislocation structures would form within the material. These severely hinder subsequent dislocation motion, making it difficult for dislocations to initiate and glide to accommodate deformation, ultimately leading to reduced ductility. Concurrently, the nanograins and nanotwins induced by LSP drastically shorten the mean free path for dislocation slip. This prevents dislocations from accumulating and moving effectively within the nanograins, resulting in a decline in plastic deformation capability.

However, recent studies have reported that LSP can simultaneously improve both strength and toughness, suggesting a favorable strength–toughness synergy. Lv et al. [75] demonstrated a 14.3% increase in yield strength and an 18.3% increase in elongation-to-failure for LSP-processed TC4 alloy (Figure 10d). Guo et al. [64] conducted comparative analysis of fracture surface morphologies, showing untreated specimens with small and shallow dimples (Figure 10e), whereas LSP-treated samples have a larger size and elongated dimples (Figure 10f).

The mechanisms responsible for LSP-induced toughness enhancement can be summarized as follows: (i) Ductility relies on the material’s ability to store dislocations and facilitate dislocation nucleation [76,77]. During LSP, the strain rate reaches its maximum at the material surface and attenuates with increasing depth. The steep strain gradient between the surface and matrix leads to the accumulation of geometrically necessary dislocations, which increases the upper limit of allowable dislocation density in the subsurface region. This, in turn, provides space for dislocation accumulation during tensile deformation [78,79]. (ii) The formation of a high density of nanotwins not only blocks dislocation motion but also provides nucleation sites for dislocations and subgrain boundaries [80], thereby enhancing the material’s ductility.

## 5. Surface Roughness

**Figure 11:**
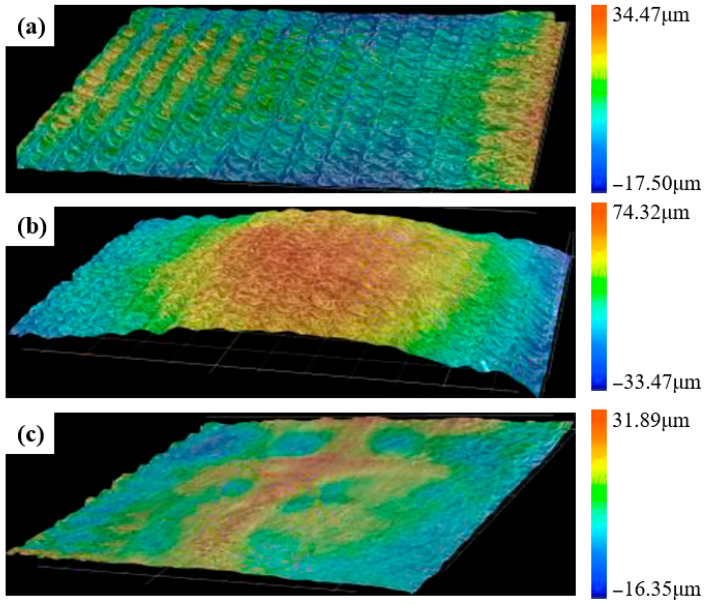
Three-dimensional morphology of samples treated by different laser shock processing: (**a**) without LSP treatment; (**b**) LSP treatment; (**c**) LSWP treatment. Reprinted with permission from [85].

During laser impact testing, overlapping laser spots generate periodic texture variations. The resulting boundary effects between adjacent spots produce complex topographical features that inherently increase surface roughness [81]. Tong et al. [82] investigated the surface roughness of samples under varying laser energy densities and found that LSP significantly altered the surface morphology. Compared with untreated samples (Figure 11a), the LSP-treated surface exhibited a 33% increase in arithmetic mean roughness at 6J laser energy (Figure 11b). Maawad et al. [83] reported that the fatigue strength of Ti-54M titanium alloy decreased after LSP treatment, primarily attributable to heightened roughness. Surface asperity peaks and valleys act as stress concentration sites and preferential crack initiation nuclei, thereby accelerating fatigue crack formation and reducing fatigue life [39].

To overcome this issue, Dai et al. [84] proposed the laser shock wave planishing (LSWP), which used a high dynamic-yield-strength contacting foil between the absorber layer and workpiece. The smooth surface of the contact film can planish the rough surface of the workpiece so as to reduce the workpiece surface roughness. Wu et al. [85] applied this technique on TC4 titanium alloy (Figure 11c), achieving a substantial reduction in areal surface roughness Sa from 14.1 μm to 4.21 μm compared to conventional LSP, coupled with a 63.78% enhancement in fatigue life. Subsequently, Wang et al. [86] introduced laser surface imprinting (LSI) based on LSWP, wherein specific geometric patterns are etched onto the metallic contact film. Under high-energy laser irradiation, these patterns regulate the material’s plastic flow, enabling precise control of surface morphology. Compared with LSWP, LSI further reduced roughness by approximately 9.4% and enhanced surface hardness due to micro-texturing-induced plastic deformation.

## 6. Coupling Mechanisms for Fatigue Performance

Indeed, the enhancement in the fatigue performance of titanium alloys after LSP arises not from any single factor, but from the synergistic interplay of multiple elements. Bai et al. [87] observed that micro–nanostructures induced by LSP—such as dislocations accumulated at grain boundaries and nanograins—serve as effective barriers to crack propagation, resulting in a more tortuous crack path in TC4 titanium alloy. Simultaneously, the compressive residual stresses introduced by LSP significantly reduce the crack growth rate. He et al. [88] utilized the coupling among compressive residual stress, nanostructured features, and surface roughness to optimize processing parameters (e.g., overlap ratio and laser scanning path). This strategy not only ensured the introduction of high-magnitude compressive residual stress but also maximized the uniformity of the near-surface microstructure. The enhanced uniformity, in turn, enabled the residual stress to alleviate stress concentrations induced by surface roughness, synergistically leading to a substantial improvement in fatigue life. Tang et al. [89] employed advanced simulation techniques, such as a three-dimensional phase-field model, to numerically couple the LSP-induced residual stress field with fatigue crack initiation and propagation processes. This approach enabled more accurate prediction of the material’s fatigue life and facilitated optimization of the strengthening effect.

To provide a more intuitive analysis of the coupled effects among various LSP-induced factors, this review consolidates key findings from multiple studies into a unified framework, as presented in Table 1. Drawing from the compiled data, it is evident that the majority of studies report a significant enhancement in the fatigue life and strength of titanium alloys following LSP treatment. This substantial increase underscores the prevailing consensus that the observed improvement predominantly results from the synergistic interaction of multiple factors. LSP is fundamentally a thermal–mechanical coupling process arising from laser–material interactions. The mechanical characteristics—such as the pressure distribution and peak value of the shock wave—and the associated thermal effects are jointly governed by key process parameters including laser energy, pulse width, and spot geometry, and so on. The complex coupling of these parameters plays a critical role in determining the resulting residual stress field and microstructural evolution. Given the multitude of influencing factors and the intricacy of their coupling mechanisms, further dedicated investigations remain essential to uncover the underlying physical mechanisms.

## 7. Conclusions

With the continuous advancement of technology and the increasing complexity of service environments, improving the fatigue performance of titanium alloys has become increasingly critical. LSP technology, characterized by its high strain-rate plastic deformation mechanism, provides a reliable method for enhancing fatigue resistance by reconstructing residual stress fields and refining microstructures, demonstrating strong potential for industrial applications.

Optimized laser parameters can generate the enhanced compressive residual stress fields characterized by greater magnitude, improved uniformity, and extended subsurface penetration depth. These compressive stresses serve as the dominant factor for improving resistance to crack initiation and propagation. Concurrently, LSP can induce grain refinement, increase dislocation density and promote deformation twinning activation. The enhanced hardness and yield strength result from the resultant microstructural alterations. From a nanoscale perspective, the enhancement or reduction in fracture toughness of titanium alloys following laser shock processing can be attributed to whether sufficient space exists within the grains to accommodate dislocation motion. Furthermore, the laser spot boundary effect can increase surface roughness, limiting the overall performance improvement achievable through LSP.

Although a general consensus exists on the enhancement of fatigue life by LSP, there are still several key issues that require in-depth exploration, especially in relation to the complex nanostructures: (i) Intrinsic mechanism of the nanostructure nucleation, deformation and interaction; (ii) A disconnect persists between experimental observations and theoretical modeling in LSP research. Developing theoretical models that can quantitatively correlate LSP-induced multiscale structural modifications with fatigue performance is essential for optimizing process parameters and accurately predicting and controlling the material’s fatigue behavior; (iii) Development of synergistic “LSP+” hybrid process, which can overcome the limitation of conventional single-stage treatment in tailoring variant nanostructures. A complementary understanding of fatigue enhancement mechanism is conducive to the development of high-performance titanium alloys and the application of laser shock strengthening technology.

## Figures and Tables

**Table 1:** Summary of coupling mechanisms on titanium alloy fatigue performance by LSP.

Materials	Methods	Peak Compressive Residual Stress	Nanostructure Characteristic	Crack Initiation Site	Surface Roughness	Fatigue Life (FL)/ Fatigue Strength (FS)	Ref.
TC4	LSP	581 MPa	DTs, Nanotwins	-	-	+238% (FL)	[31]
TC4	LSP	646 MPa	HDDs, Nanotwins	Altered	-	+114.95% (FL)	[41]
TC4	LSP	161.2 MPa	-	Altered	+188.76%	−22.22% (FS)	[42]
TC4	LSP	750 MPa	HDDs, Nanocrystals	-	-	+6.25% (FS)	[46]
TC4	LSP	260 MPa	HDDs	-	-	+126% (FL)	[90]
TC4	LSP + SP	728 MPa	DTs, HDDs, Nanotwins	-	-	+930% (FL)	[32]
TC4	LSP + SP	843.2 MPa	HDDs, Nanocrystals	-	-	+35% (FS)	[91]
TC4	AM + LSP	598 MPa	-	Altered	-	+137% (FL)	[28]
TC4	AM + LSP	-	HDDs, Nanotwins	-	-	+16.67% (FS)	[45]
TC4	HFEP-LSP	906 MPa	DTs, HDDs	-	-	+176.4% (FL)	[7]
TC4	LSP	354 MPa	-	-	+121.35%	+4.38% (FL)	[85]
	LSWP	337 MPa		Altered	−33.91%	+63.78% (FL)	
TC21	LSP	550 MPa	HDDs, Nanotwins	Altered	-	+161% (FL)	[92]
TC17	LSP	639 MPa	HDDs	Altered	+104.74%	+272.55% (FL)	[88]
TC17	LSP	460 MPa	HDDs, SFs	-	-	+330% (FL)	[93]
TC17	LSP	460 MPa	HDDs, SFs	Altered	-	+50% (FL)	[94]
TC17	AM + LSP	727 MPa	HDDs, Nanocrystals	-	-	+23.6% (FS)	[17]
TC11	LSP	589.2 MPa	HDDs, Nanocrystals	Altered	-	+22.8% (FS)	[20]
TC11	LSP	645 MPa	-	Altered	+61.7%	+116.09% (FL)	[95]
TC6	LSP	556.2 MPa	HDDs, Nanocrystals	-	-	+20.1% (FS)	[96]
TB10	LSP	247 MPa	HDDs, SFs, Nanocrystals	-	-	+40.2% (FL)	[52]

Abbreviations: LSP, laser shock peening; SP, shot peening; AM, additive manufacturing; HFEP-LSP, high-frequency electropulsing-assisted laser shock peening; LSWP, laser shock wave planishing; DTs, dislocation tangles; HDDs, high-density dislocations; SFs, stacking faults.

## Data Availability

No new data were created or analyzed in this study. Data sharing is not applicable to this article.
